# Storage stability study of metronidazole and hydroxymetronidazole in chicken eggs by liquid chromatography tandem mass spectrometry

**DOI:** 10.1016/j.fochx.2024.102087

**Published:** 2024-12-10

**Authors:** Mikhail Vokuev, Artem Melekhin, Anastasia Frolova, Anton Bairov, Igor Rodin, Victor Tishchenko

**Affiliations:** a Department of Chemistry, Lomonosov Moscow State University, 119991 Moscow, Russia; b Federal Centre for Animal Health, 600901 Vladimir, Russia

**Keywords:** Metronidazole, Hydroxymetronidazole, Liquid chromatography, Tandem mass spectrometry

## Abstract

Metronidazole (MNZ) is one of the most commonly used antibiotics in the food industry. High levels in food can lead to the development of antimicrobial resistance in humans, so it is important to monitor its levels in food. In the context of legal proceedings, it is frequently necessary to re-examine samples after an extended period of time. It is therefore crucial to ensure that the analytes in question do not degrade during the storage period. In this study, HPLC-MS/MS approach was validated and used to analyze levels of MNZ and its hydroxy metabolite in chicken eggs during storage in the refrigerator (+4 °C) and in the freezer (−20 °C) for 3 months. An analyte solution was administered to hens to obtain eggs containing MNZ and hydroxymetronidazole (MNZ-OH). The dependence of the analyte content in eggs as a function of time after sampling was also investigated.

## Highlights

• A dosage of metronidazole was administered via intramuscular injection to live hens.
• Eggs were collected for 11 days following the administration.
• The proposed approach for the quantitative analysis of analytes in eggs was validated.
• The rate of analyte degradation in eggs was monitored during storage at +4 and −20 °C.
• The analytes are stable in eggs at −20 °C for at least 3 months.

## 1 Introduction

The demand for high quality animal source foods with a long shelf life is increasing from year to year (Gautron et al., 2022; Henchion et al., 2021). Stable quality of products depends on the hygienic conditions of production and on the condition of animals entering the slaughterhouse. Poultry meat and eggs are considered to be important sources of essential nutrients and a major contributor to the elimination of dietary mineral deficiencies. These products include essential amino acids, vitamins K, B7 (biotin) and B12, folic acid, and iodine.

Chicken meat and eggs provide high quality protein. Poultry meat, unlike other meats, is high in healthy monounsaturated fats. Eggs, in turn, have the highest net protein utilization (NPU) index, which reflects the protein quality (Gilani & Lee, 2003). Chicken production ranges from specialized intensive units, where it was originally established to produce either meat or eggs, to backyard systems that raise dual-purpose chicken breeds for local consumption and sale, up to large-scale industrialized enterprises (Henchion et al., 2021).

In terms of maintaining sustainable production of animal-based foods, antibiotics are widely used for the prevention and treatment of infectious diseases and as food additives to stimulate growth (Hong et al., 2022; Xu et al., 2021). Antimicrobial and chemotherapeutic drugs in animal agriculture are used for therapeutic purposes, while the targeted action of antibiotics also increases nutrient utilization in the diet, thereby reducing feed costs. For example, 80 % of antibiotics sold in the United States are used in livestock and poultry production, mainly as growth promoters or to prevent disease outbreaks due to inadequate housing and husbandry conditions (huddling of animals or poultry, or lack of proper sanitation) (Martin et al., 2015).

The presence of residues of antimicrobial drugs in food of animal origin poses a serious risk to human health due to the manifestation of pronounced toxic and allergic properties, as well as the transfer of antibiotic-resistant bacteria to humans, serving as a potential source of zoonotic or non-communicable diseases. Eggs are considered to be one of the most likely food products to contain antibiotics, and are most likely to be found in (Lima et al., 2023; Owusu-Doubreh et al., 2023). The ingestion of antibiotics in an uncontrolled manner with foodstuffs can result in the emergence of resistance to certain drugs. Unauthorized use of such drugs in livestock products without labelling their use can be considered as a form of falsification.

Drugs of the 5-nitro are usually used in poultry production for the treatment and prevention of diseases of bacterial etiology (aeromonosis, cryptobiosis, spironucleosis, balantidiosis), as well as in other diseases complicated by anaerobic and protozoan infections in poultry (laying and broiler hens and when breeding chickens). Metronidazole is considered the first 5-nitroimidazole-based drug and was approved for medical use in 1960 (Davis & Papich, 2014). Subsequently, tinidazole, ornidazole, secnidazole, etc. were developed, including ternidazole for topical use. Secnidazole is characterized by a longer half-life, which allows the use of a single dosing regimen instead of multiple dosing (Thulkar et al., 2012).

To date, the most commonly used derivatives of nitroimidazoles (NIMs) are dimetridazole (DMZ), ipronidazole (IPZ), metronidazole (MNZ), and ronidazole (RNZ) consisting of an imidazole ring with a nitro functional group in the fifth position and at least one substituent. The presence of the latter determines antibiotic and anticoccidial activities based on the biochemical reduction of the 5-nitro group by intracellular transport proteins of anaerobic organisms and protozoa. The reduced 5-nitro group interacts with the deoxyribonucleic acid (DNA) of a bacterial cell, inhibiting nucleic acid synthesis, which leads to the death of the microorganism (Bury-Moné, 2014).

However, these 5-nitroimidazole-based antibiotics have serious implications on human health due to their carcinogenic and mutagenic properties (Li et al., 2022). To prevent of their presence in food of animal origin, the use of NIMs is prohibited in many countries such as the United States and the European Union and no maximum residue limits (MRLs) are established. In China the MRL for DMZ and MNZ in poultry muscle is 50 μg/kg (Yang et al., 2014).

NIMs are rapidly metabolized by oxidation of the side chain of the imidazole ring at C-2 position to form hydroxy metabolites which also interfere with normal body functions (Tamošiūnas & Padarauskas, 2009). The metabolites to be monitored are: hydroxymetronidazole (MNZ-OH), 2-hydroxymethyl-1-methyl-5-nitroimidazole (HMNNI) - product of both RNZ and DMZ metabolism, and 1-methyl-2-(2′-hydroxyisopropyl)-5-nitroimidazole (IPZOH) for IPZ (Tamošiūnas & Padarauskas, 2009; Tölgyesi et al., 2012).

A large variety of sample preparation techniques and analytical methodologies for the determination of NIMs are being developed for the control of presence of their residues in various products of animal origin, biological and environmental samples (Mahugo-Santana et al., 2010). Recent studies indicate the relevance of eggs as promising target matrices for antibiotic residues' analysis due to the homogenous distribution of analytes and their stability compared to poultry muscle where a rapid decrease of NIMs' concentration is shown during storage above 4 °C (Cronly et al., 2009).

The most commonly used technique for analyte concentration in egg-based matrices is solid phase extraction (SPE) because of its relative simplicity and the possibility of combining it with different determination methods (Cronly et al., 2009; Piatkowska et al., 2016; Semeniuk et al., 1995; Tamošiūnas & Padarauskas, 2009; Tölgyesi et al., 2012; Tölgyesi et al., 2017). Acetonitrile is the preferred extraction solvent for the isolation of nitroimidazoles from egg matrices, as the use of ethyl acetate results in the formation of stable emulsions (Mahugo-Santana et al., 2010). In addition, hexane can be used for sample clean-up if fat removal is required (Fraselle et al., 2007; Shen et al., 2003).

After sample preparation, a highly selective and accurate determination is performed, usually based on chromatography combined with mass spectrometry. Liquid chromatography mass spectrometry (LC-MS) demonstrates its versatility in addressing diverse research objectives within pharmacological studies. It is a widely applicable analytical tool used at various stages of research, from uncovering therapeutic mechanisms to ensuring drug safety, as evidenced by its use in both exploratory analyses and safety-focused studies (Lang et al., 2023; Olaitan et al., 2023). The latter is particularly evident in its extensive application for detecting antibiotic residues in various sample matrices. For the ionization of NIMs atmospheric pressure chemical ionization (APCI) and electrospray ionization (ESI) interfaces are often used, and the determination is usually performed under positive ionization conditions (Mahugo-Santana et al., 2010).

Liquid chromatography-tandem mass spectrometry (LC-MS/MS) with sample preparation and clean-up allows the qualitative and quantitative determination of 5-nitroimidasols in both eggs (Cronly et al., 2009; Daeseleire et al., 2000; Sams et al., 1998; Z. Zhang et al., 2017) and egg powder (Mohamed et al., 2008; Mottier et al., 2006). Appropriate extraction techniques combined with LC-MS/MS determination allow significantly lower limits of quantification of the analytes studied. Ion suppression negatively affects the results of mass spectrometry determination, hinders accurate MS quantification and is caused by matrix effect. The latter can be eliminated in several ways, including the use of deuterated standards of NIMs (Mitrowska et al., 2010).

Routine approach on study animal-derived foods in terms of non-recommended drugs identification contain certain conditions of operating procedures. A fairly wide acquaintance with the literature shows changes in analytical methods and their enhancement in relation to biological samples of animal origin (F. Li et al., 2025; Rabea et al., 2024; Mo et al., 2024; Lima et al., 2024). In recent years a considerable amount of work has been done in sample preparation, extraction of target analytes of veterinary drugs and their accurate identification however both the problem of storage conditions and study of products of animals exposed to drug interactions (Youssef et al., 2013) has not received all the attention it deserves. Sample handling and preservation has not thoroughly studied compared to purification, separation and determination techniques. Meanwhile, to obtain reliable results a transfer of drug residues and their metabolites in animal source foods and either the impact of storage conditions on the “quality” and suitability of final sample are obligatory to understand and should be taken into account to ensure human food safety by confirming the alleged irrational use of antibiotics in agriculture.

In present work determination of MNZ and its related hydroxy metabolite in eggs laid by exposed poultries was performed and variations of their content in time and in different storage conditions are estimated.

## 2 Materials and methods

### 2.1 Chemicals

Reference standards of metronidazole, hydroxymetronidazole, metronidazole-D_3_ and hydroxymetronidazole-D_2_ of high purity grade (≥95.0 %) were purchased from sigma Aldrich (St. Louis, MO, USA) and Witega (Berlin, Germany). Stock 200 mg L^−1^ standard solutions were prepared by dissolving in methanol. Sonication was applied to ensure complete dissolution. Solutions of internal standards were prepared in a similar way. Three-level standard working solutions were prepared by combining of each stock solution in methanol to achieve the desired concentration of 10, 100 and 1000 μg L^−1^. The working solution of internal standards concentration of 1000 μg L^−1^ were prepared in a similar way. The standard stocks and working solutions were stored at −20 °C in brown glass to prevent degradation. The stock and diluted solutions validity was 12 and 6 months, respectively

HPLC-grade methanol, acetonitrile, formic acid, *n*-hexane and water were obtained from Fisher Scientific Inc. (Pittsburgh, PA, USA).

### 2.2 Collection of egg and poultry samples

The experiment required the collection of samples of chicken eggs. For this purpose, five healthy laying hens of a similar size were acquired (with a body weight of 1.9–2.1 kg). Samples of “clean” eggs were collected before the drug was administered. To confirm the absence of drug residues, the eggs were tested twice, with a one-week interval between each verification. The “clean” samples (blank) were later used to build calibration curves.

Metronidazole was administered intramuscularly in an amount of 10 mg per chicken for 5 days. Two days after discontinuation of the drug, egg sampling was started. Thus, eggs were sampled on days 3, 5, 7, 9 and 11 after antibiotic administration to hens. All samples collected on the 3rd, 5th, 7th, 9th and 11th day of the study after parenteral administration of the drug were mixed well, homogenized and stored at a temperature no higher than −80 °C prior to the main storage experiments.

### 2.3 Sample preparation

Contents of the analyzed egg were separated from the shell and mixed well. 100 μL of internal standard was added to 1 g of the obtained sample. The sample was mixed and remained for half an hour. Then 3 mL of acetonitrile was added and vortex-mixed (mixed in the shaker) for 15 min and centrifuged at 4000 rpm at 4 °C for 15 min. Organic phase was placed into another tube and evaporated under the nitrogen flow at 40 °C, then dissolved in a 1 mL of mobile phase mixture with solvent ratios of methanol and acetonitrile been equal to 95 and 5 (containing methanol and acetonitrile in ratio of 95:5). After that 4 mL of hexane was added, the probe was mixed and centrifugated for 15 min. Organic hexane phase was removed and the resulting solution was filtered through a 0.25 μm Chromafil Xtra filter membrane (Macherey-Nagel, Germany) into LC vial for the HPLC-MS/MS analysis.

### 2.4 LC–MS/MS conditions

For the chromatographic separation a Shimadzu HPLC Nexera X2 liquid chromatograph equipped with a binary pump and an autosampler was used. Separation was carried out using an Acclaim 120 C18 column (100 × 2.1 mm, 3.0 μm, Thermo Scientific, USA) in a gradient elution mode. The column and the autosampler were maintained at 40 and 15 °C respectively during operation. The analytes were separated using the mobile phase containing 0.5 % formic acid in water (eluent A) and 0.5 % formic acid in the mixture of acetonitrile / methanol 1:1 (eluent B). The gradient program was as follows: 0–0.2 (5 % B), 0.2–5.0 min (5 % B - 95 % B), 5.0–6.0 min (95 % B - 5 % B), 6.0–6.1 min (5 % B), 6.1–7.0 min (5 % B). The mobile phase flow rate was 0.3 mL min^− 1^. The injection volume was 10 μL.

A triple quadrupole mass spectrometer LCMS-8060 Shimadzu was configured to collect data in the multiple reaction monitoring (MRM) mode. The following optimal ESI-MS/MS conditions were set: ion source nebulizer gas, 3 L min^−1^; ion source gas for drying solvent, 10 L min^−1^; heating gas, 10 L min^−1^; interface temperature, 300 °C; desolvation line temperature, 250 °C. MRM conditions, collision energy (CE), Q1 Pre Bias (V), and Q3 Pre Bias (V) were first optimized for each analyte by injecting solutions of the veterinary drug standards prepared in the mobile phase. Characteristic molecular ions were selected as precursor ions and two product ions were monitored for each compound. The most intense MRM transition was chosen for quantification and was monitored together with the second transition for confirmation. MRM parameters for analytes and internal standards are given in Table 1.

**Table 1:** Mass spectrometry parameters for MRM mode.

Compound	Precursor Ion	Product Ion	Q1, V	CE, V	Q3, V
Metronidazole	172.1	128.1	−11	−17	−12
		82.1	−11	−25	−14
Hydroxymetronidazole	188.1	123.1	−12	−13	−12
		126.1	−12	−17	−12
Metronidazole-D_3_	175.1	131.1	−11	−17	−12
Hydroxymetronidazole-D_2_	190.1	125.1	−13	−15	−12

### 2.5 Method validation

The method for the determination of MNZ and MNZ-OH in eggs has been validated in order to improve the reliability for the following parameters: specificity, linearity, accuracy, precision, limit of detection (LOD) and limit of quantification (LOQ). To confirm specificity, blank eggs containing no target analytes were used and no interfering peaks on the retention time of the target analytes were observed in the chromatograms of these eggs. To establish the calibration curve, MNZ and MNZ-OH were spiked into blank eggs at different concentrations (from 0.5 to 350 μg/kg) and sample preparation was carried out. The ratio of the peak areas of the analytes and IS was then plotted as a function of concentration. Accuracy and precision were evaluated using quality control samples (QCs) prepared by spiking blank eggs with MNZ and MNZ-OH at three concentrations (1, 5, 250 μg/kg) different from the calibration levels. Each QCs was analyzed 6 times in a day. Concentration of QCs was determined by using calibration curve. Accuracy was calculated as the ratio of calculated concentration to actual concentration.

$$\;\mathrm{Accuracy}\;\left(\%\right)=\frac{c}{c_{0}}*100$$

where c is found concentration of an analyte in sample, c_0_ is actual concentration.

The precision was assessed as relative standard deviation (RSD) of 6 replicates for each QCs.

$$\mathrm{RSD}\left(\%\right)=\frac{\sigma }{c}*100$$

where σ is the standard deviation, c is the mean value of the found analyte concentrations in the sample.

LOD and LOQ were evaluated by signal-to-noise ratios of 3 and 10, respectively. For this purpose, the analytes at low concentrations were spiked to eggs, and the samples were subsequently prepared and analyzed. The concentrations at which signal-to-noise ratio was equal to 3 and 10 were selected as LOD and LOQ, respectively. The values of matrix effects (ME) were calculated using the correlation coefficients for calibrations obtained on eggs and water under sample preparation conditions according to the formula

$$\mathrm{ME}\left(\%\right)=\frac{A}{B}*100$$

where A is the slope coefficient of the calibration curve in water, and B is the slope coefficient of the calibration curve in egg.

## 3 Results and discussion

### 3.1 HPLC-MS/MS conditions

**Fig. 1:**
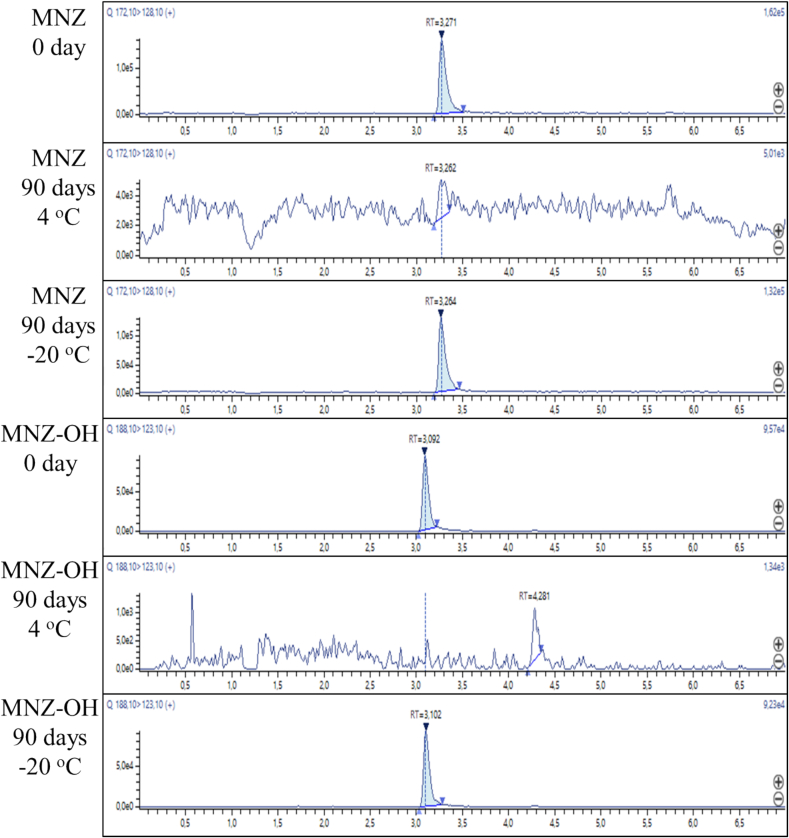
MRM chromatogram of egg samples. The upper three chromatograms correspond to MNZ and the lower three correspond to MNZ-OH.

In the process of chromatographic condition optimization different compositions of mobile phases were tested. Pure water, aqueous solutions of formic acid (0.1 and 0.5 % vol.) and ammonium formate (10 mM and 40 mM) were used as an aqueous eluent. The organic phases tested were acetonitrile, methanol and a 1:1 mixture of these, including the addition of formic acid. Reversed-phase chromatography columns of different lengths and sorbent sizes such as Acclaim 120 C18 (100 × 2.1 mm, 3.0 μm, Thermo Scientific, USA), Acclaim 120 C18 (150 × 2.1 mm, 2.2 μm, Dionex, USA) and EclipsePlusC18 RRHD (100 × 2.1 mm, 1.8 μm, Agilent, USA) were proven. Based on analysis of key parameters such as analyte peak shape, retention time, selectivity and sensitivity, the Acclaim 120 C18 100 mm was identified as the preferred column. The addition of formic acid to the eluent enhanced the sensitivity of the determination of metronidazole and hydroxymetronidazole by registering positive ions resulting from the protonation of the analytes in an aqueous environment. As a result, a 0.5 % aqueous formic acid solution and a 0.5 % solution of formic acid in methanol-acetonitrile mixture (1:1) were chosen as the mobile phases. The rate of change in the mobile phase composition was also varied. As a result, a 7-min gradient with a flow rate of 0.3 mL min^−1^ was proposed. Fig. 1 shows the MRM chromatograms of egg samples after 90 days of storage at 4 and − 20 °C, compared to samples prior to storage.

**Fig. 2:**
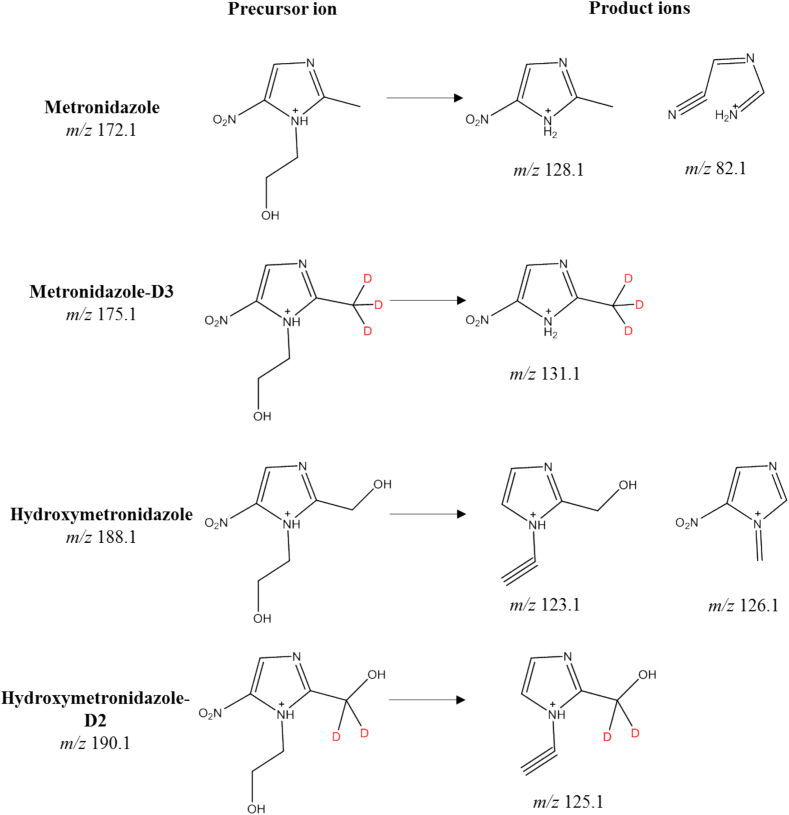
Proposed structures of precursor and product ions for MNZ, MNZ-OH, and IS.

As part of the optimization process for MS detection, precursor and product ions were initially selected with the highest intensities using individual standard solutions. Protonated molecules [M + H]^+^ were selected as precursor ions for all analytes. As a result of collision induced dissociation (CID), ions with mass-to-charge ratios of 128 and 82 were formed from the protonated metronidazole, while ions with *m*/*z* of 123 and 126 were formed from protonated hydroxymetronidazole. Considering the nitrogen rule, as well as the fact that the initial analyte molecules consist of hydrogen, oxygen, carbon and nitrogen, the proposed structural formulas of the product ions are shown in Fig. 2. Ions with even-numbered m/z have an odd number of nitrogen atoms in their composition, and ions with odd m/z contain an even number of nitrogen atoms. This rule should be adjusted for deuterated analogues. Parameters such as Q1/Q3 Pre Bias and CE were optimized for the selected ion transitions in MRM mode. The optimal values to maximize sensitivity are shown in Table 1. Thus, the optimal HPLC-MS/MS conditions were chosen for sensitive quantitative analysis of MNZ and its metabolite MNZ-OH in eggs.

### 3.2 Optimization of sample preparation conditions

An organic solvent was employed to extract analytes from eggs. The addition of solvents such as acetonitrile and methanol also precipitated proteins from the sample. Acetonitrile and methanol were compared with respect to the recovery of MNZ and MNZ-OH from eggs. Consequently, there was no significant difference between the two solvents, so our decision was to use acetonitrile. After extraction with acetonitrile, the organic fraction was centrifuged at 4 °C, evaporated under nitrogen and redissolved in 1 mL of a 95:5 mixture of mobile phases.

**Fig. 3:**
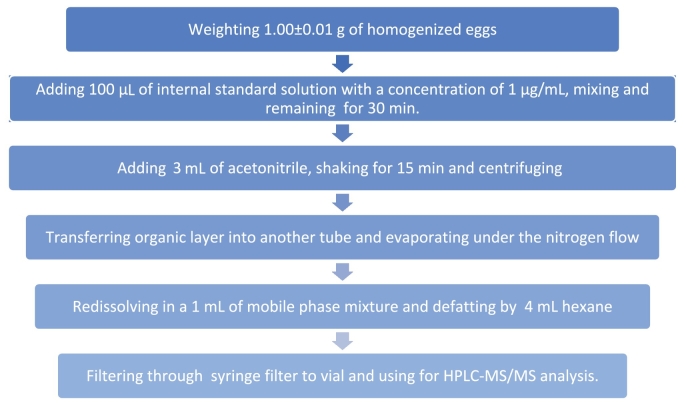
The optimized sample preparation scheme.

It should be noted that eggs also contain a significant amount of fat, and that a further purification process is typically employed to remove this. Liquid-liquid extraction (LLE) with hexane, which dissolves the fats, has been used for additional purification. The impact of varying volumes of hexane (between 1 and 5 mL) on ME value was investigated. Up to a hexane volume of 3 mL, ME was found to significantly reduce the sensitivity of analyte determination. As no significant difference was found between 4 and 5 mL of hexane, the use of a volume greater than 4 mL was not appropriate. Double LLE with hexane was also tested. ME for MNZ was 95 and 98 % for single and double LLE, respectively. ME for MNZ-OH was 101 and 99 % in the case of single and double LLE, respectively. Therefore, it was determined to utilize a single LLE procedure by using 4 mL hexane to remove lipids. The optimized sample preparation scheme is shown in Fig. 3.

### 3.3 Method validation

Linearity, LOQ, precision, recovery, and ME of the developed method were determined. MNZ, MNZ-OH and IS were spiked to blank eggs, and sample preparation was performed. The resulting extracts were analyzed to plot calibration curves. The ratio of the analyte and IS areas was plotted on the Y-axis, and the analyte concentration was plotted on the X-axis. The correlation coefficients for MNZ and MNZ-OH, found by the least squares method, were greater than 0.99, indicating acceptable linearity of the calibration curves. LOQ was 0.5 μg/kg. Accuracy did not exceed 15 % for all QCs. Intra- and inter-day RSD did not exceed 15 % for QC samples in three replications. ME and recovery were estimated at three concentration levels using QC samples. Recovery for MNZ and MNZ-OH was in the range from 98 to 102 %, which confirms full extraction of the analytes from eggs. The mean ME of MNZ and MNZ-OH were 98 % and 101 %, respectively. Despite good ME and recovery, deuterated analogs of MNZ and MNZ-OH were used in the work to increase the reliability of the results obtained. The quantitative parameters described are in Table 2.

**Table 2:** Quantitative parameters of the approach.

Analyte	R^2^	Spiked level, μg kg^− 1^	Recovery, %	Intra-day RSD, %	Inter-day RSD, %	LOQ, μg kg^− 1^	ME, %
MNZ	0.999	1	99	12	15	0.5	98
		5	100	10	12		
		250	101	6	7		
MNZ-OH	0.998	1	102	11	13	0.5	101
		5	98	10	11		
		250	99	6	8		

### 3.4 Analysis of eggs from hens exposed to MNZ

In recent work storing temperature for collected egg samples from hens pretreated with MNZ was studied. Content levels of MNZ-OH as the MNZ metabolite were also monitored. Content measurement of MNZ and MNZ-OH in collected eggs started after period of 3 days following withdrawal of the antibiotic in accordance with the research study (Youssef et al., 2013) where MNZ was detected in egg samples after the third day of antibiotic administration. In spite the fact that a period of deposition and accumulation of antibiotics in hens and accordingly in eggs may vary due to the nature of egg formation and physiology of a hen (Owusu-Doubreh et al., 2023) – the drug accumulation pattern is similar both in case of oral and parenteral medication (Cybulski et al., 1996) and begins after the first dose is administrated.

**Fig. 4:**
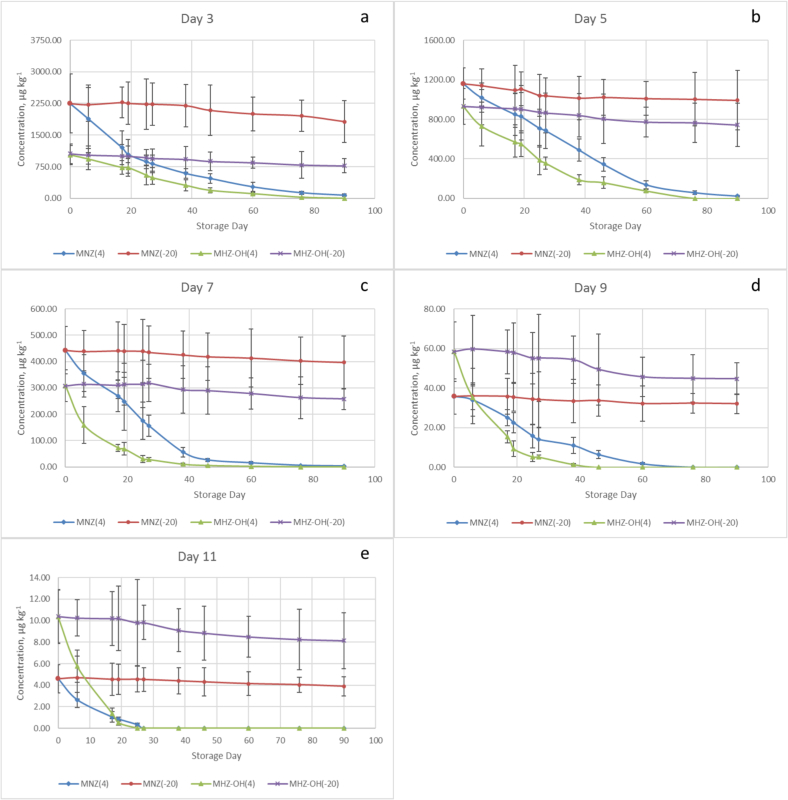
Dependences of MNZ and MNZ-OH content in eggs on storage time at +4 and − 20 °C. Samples of eggs were obtained on the 3rd (a), 5th (b), 7th (c), 9th (d) and 11th (e) days following the administration of MNZ to the hens.

For food analysis, it is necessary to select the optimal storage conditions for objects under study. It is essential that the stored control samples retain their original characteristics and composition to ensure the reliability of the analysis and to facilitate rechecking of results in other laboratories in borderline situations and legal proceedings. Two storage temperatures were selected for storing egg samples: 4°С and − 20°С for all five points with different concentrations of MNZ and MNZ-OH (different days after MNZ administration). For all concentration levels, the storage kinetics graphs are presented in Fig. 4.

After a month of storage of egg samples at 4 °C, the concentration of MNZ decreased by an average of 60 %, the concentration of MNZ-OH by 75 %. In the case of the sample with the lowest concentrations (Fig. 4e), after a month of storage at 4°С, peaks of MNZ-OH and MNZ were not observed, while after a period of one month during which the egg samples were stored at −20°С, the concentration remained stable for both analytes. After three months of storage at 4°С, the peak of MNZ-OH was not observed in the eggs sampled from the 5th day after the MNZ administration (Fig. 4b–e), and in the sample with the highest analyte content the concentration of MNZ-OH dropped by more than 99 %. For MNZ, the concentration fell by an average of 98 % of the initial value. After three months of storage at −20°С, the concentration of metronidazole decreased by an average of 14 %, and the concentration of MNZ-OH by 22 %. As can be seen from the results obtained when detecting MNZ and MNZ-OH in the egg sample, further storage of this sample should be carried out at −20°С for better preservation of the initial composition in case of a rechecking the results of the determination.

The starting points on the graphs correspond to MNZ and MNZ-OH content in eggs sampled on days 3–11 after the antibiotic administration to hens, prior to the storage experiment. The highest MNZ content was observed on the 3rd day after MNZ administration and was equal 2.3 mg kg^−1^. On the 5th and 7th days of egg sampling the concentration levels had a considerable change and fell almost twice from the moment of the last probe obtaining and stood at nearly 0.45 mg kg^−1^. Then followed a dramatic decrease with the value of 36 μg/kg ending at the point of 4.6 μg/kg on the 11th day of egg laying after MNZ administration. Variance in starting levels of MNZ could be explained by a complicated nature of its incorporation into eggs. However, metronidazole is known to have a minimal plasma protein binding in hens thus the drug is easily absorbed and rapidly distributed to various tissues reaching ovaries where the egg formation process occurs. Nevertheless, after passing through the stomach wall, MNZ is protonated in the acidic gastric juice, and this form cannot cross cell membranes back into plasma.

The slow rate of yolk formation in the oviduct, which results in slow removal and elimination of target analytes from it, and a relatively short period during which an albumen is formed, provide an opportunity to sample eggs for further analysis in a day using highly selective analytical methods such as LC-MS, without losing vital information. So, in spite the fact of substance's 24 h elimination from hens (Cybulski et al., 1996) we see drug resides even on the 11th day of egg-laying after the termination of the MNZ intake. This may also be a result of the structural similarity with other veterinary drug of the 5-nitroimidazole type, DMZ, characterized by lipophilic nature and therefore allowing its penetration into eggs. According to studies (Posyniak et al., 1996) a lipid solubility of the latter is an important factor in the persistence of residues both in yolk and albumen.

It is interesting to note that the tendency of maintaining of concentration levels both for MNZ and MNZ-OH remains through all the period of study (90 days) for each probe being investigated in a certain day of egg sampling in case of the storage temperature of −20°С while at 4°С the content of the target analytes gradually decreased (Fig. 4). It can be inferred that eggs should be stored at the lowest possible temperature in order to optimize the retrospectivity of MNZ analysis.

In animal species, metronidazole is subject to extensive oxidative and reductive metabolism. The second one may lead to toxic short-lived intermediates that bind to macromolecules in bacteria and protozoa. Variations in different factors such as temperature may affect the rate of these processes and the stability of substances in final sample. The results obtained shows that the ratio of MNZ/MNZ-OH concentrations in eggs stored at −20°С remains almost unchanged from 0 to 90 day of the further study. The contents of both decreased slowly. The ratio of MNZ/MNZ-OH at 4°С, on the other hand, varies during the investigation period which can be related to the process of their intensive degradation differing in time. The tendency of predominance of MNZ content over MNZ-OH remained for both cases. In contrast, for the 9th and 11th day after MNZ exposion it was found that the levels of MNZ-OH are slightly higher than those corresponding to MNZ.

The stability of veterinary drugs, such as MNZ and MNZ-OH, in food products like chicken eggs is crucial for assessing their persistence over time, including the potential allergenic properties of their residues. The analysis of food safety and allergenicity fits within the broader framework of understanding how processing affects allergens and how drug residues persist in food (Y. Zhang et al., 2024). Understanding how different treatments, whether through food processing or storage, can impact the safety of food is essential for managing risks related to allergens and drug residues.

## 4 Conclusion

The proposed HPLC-MS/MS approach was validated and subsequently applied to the determination of MNZ and MNZ-OH in eggs obtained from hens that had been injected with MNZ. A slight alteration in MNZ and MNZ-OH concentration was observed in eggs stored at −20 °C for 3 months. Concurrently, the analysis revealed a notable decline in the analyte contents in the eggs stored at 4 °C, even during a 30-day period. Furthermore, the current data indicates that both MNZ and its metabolite were present in eggs collected on 11th day following MNZ administration to hens. The obtained results permit the monitoring of egg-producing animals over extended periods. This process guarantees the delivery of precise data regarding animal food products to customers and consumers. Further research directions could include an investigation into the long-term impact of antibiotics on their presence in meat and eggs, as well as an examination of how the composition of hens' diets affects the levels of antibiotics found in meat and eggs.

## ARRIAH bioethics commission statement

The planning of animal experiments was carried out within the framework of Directive 2010/63/EU of the European Parliament and of the Council of 22 September 2010 on the protection of animals used for scientific purposes.

In order to implement the experiment on poultry in a laboratory vivarium, individual cages were isolated, in which conditions were created identical to those used for rearing (permanent access to clean water through nipple drinkers, feeding on a schedule, feeding with herbs).

Due to the fact that the experiment is impossible without the participation of animals, the number of chickens for the experiment is minimal, chickens do not experience inconvenience during work, slaughter is not implied by the experiment program, the Bioethics Commission of ARRIAH has issued permission to carry out this work No. 2024/7 dated 19 February 2024.

## CRediT authorship contribution statement

**Mikhail Vokuev:** Writing – review & editing, Writing – original draft, Visualization, Data curation. **Artem Melekhin:** Validation, Methodology, Investigation, Formal analysis, Conceptualization. **Anastasia Frolova:** Writing – review & editing, Writing – original draft. **Anton Bairov:** Validation, Formal analysis. **Igor Rodin:** Supervision. **Victor Tishchenko:** Supervision, Investigation, Funding acquisition, Conceptualization.

## Declaration of competing interest

The authors declare that they have no known competing financial interests or personal relationships that could have appeared to influence the work reported in this paper.

## Data Availability

Data will be made available on request.
